# The accuracy and consistency of public health data in Brazilian information systems: identification of gaps and challenges to be faced in a municipality in the Amazon region

**DOI:** 10.3389/fpubh.2025.1681810

**Published:** 2025-10-01

**Authors:** Diego Simeone, Andrea Laranjeira, Pedro M. R. Lopes, Vytória C. S. Nogueira, Daniele S. Sousa, Yago J. Martins, Marcus W. A. Carvalho, Indira A. L. Eyzaguirre, Marcus E. B. Fernandes, Aldemir B. Oliveira-Filho

**Affiliations:** ^1^Afya Faculdade de Ciências Médicas, Bragança, PA, Brazil; ^2^Laboratório de Ecologia de Manguezal, Instituto de Estudos Costeiros, Universidade Federal do Pará, Bragança, PA, Brazil; ^3^Associação Sarambuí, Bragança, PA, Brazil; ^4^Departamento de Ciências Exatas, Centro de Ciências Aplicadas e Educação, Universidade Federal da Paraíba, Rio Tinto, PB, Brazil; ^5^Grupo de Estudo e Pesquisa em Populações Vulneráveis, Instituto de Estudos Costeiros, Universidade Federal do Pará, Bragança, PA, Brazil

**Keywords:** epidemiologic surveillance, data collection, disease reporting, Health Policy, Sinan, DataSUS

## Abstract

**Background:**

In Brazil, health conditions of public importance are notified by municipal health departments with a standardized flow using the Notifiable Health Conditions Information System (Sinan). This information goes through a process of consolidation and transfer to DataSUS, the national system that centralizes health information in Brazil. This study assessed whether there are quantitative differences between notified health conditions through the municipal system (Sinan) and those consolidated in the national system (DataSUS).

**Methods:**

This study was based on the municipality of Bragança, located in the eastern Amazon, which plays a strategic role due to its high annual number of notifications. To identify differences between the systems, we used data provided by Sinan and retrieved from DataSUS from 2019 to 2023. We tested the absolute loss in the number of notifications across years and health conditions between the two systems. Ethical approval was not required due to the anonymous nature of the data.

**Results:**

Of the 19 health conditions identified and analyzed, 15 showed decreases between the systems, with losses reaching up to 91%. The largest discrepancies were observed for AIDS, syphilis in pregnant women, and dengue. Over the years, the loss was consistent, averaging approximately 42%.

**Conclusion:**

The differences observed between the two systems may have direct implications for the design, planning, and implementation of public health policies. Reducing these gaps urgently requires strategies such as the training of healthcare professionals, the revision of data flow processes, and investments in technologies that support system integration.

## 1 Introduction

In Brazil, notified health conditions of public importance are reported weekly by Municipal Health Departments to the Ministry of Health through a computerized system. This information is important for epidemiological surveillance and for the development of effective public policies (1, 2). To ensure a standardized flow of information, the Notifiable Health Conditions Information System (Sinan) is used, playing a key role in this monitoring. This system records information on infectious and communicable diseases, accidents involving venomous animals, interpersonal or self-inflicted violence, severe occupational accidents, and outbreaks of transmissible diseases (for more information on the system, visit https://www.gov.br/saude/pt-br/composicao/svsa/sistemas-de-informacao/sinan).

Epidemiological information on the notified health conditions and sociodemographic data of the patients is collected through individual notifications by health professionals from primary health care units and hospitals. These professionals include physicians, nurses, and public health agents. Afterwards, these data are recorded in the local system (Sinan) by epidemiological surveillance agents, which are managed by municipal health services. At this stage, information regarding the investigation of individual notifications is entered into Sinan. This includes requests for laboratory tests to confirm infectious diseases, case outcomes (confirmed or discarded, death or recovery), and outcomes of accidents involving venomous animals and severe occupational accidents. According to Sinan protocols, these records must be finalized within 60–180 days. However, even if a notification was not finalized, it must be reported weekly to the Brazilian Ministry of Health. Subsequently, the information goes through a process of consolidation and transfer to DataSUS, the national system that centralizes health information in Brazil. DataSUS consolidates the confirmed cases of notifiable conditions, continuously updating its system with the weekly data provided by Sinan. This transfer flow, although necessary for national-scale data integration, often encounters issues that result in discrepancies between the values of confirmed health conditions recorded in the local system and those effectively consolidated in DataSUS. This issue is also frequently experienced by other countries (2–4). Possible causes of these discrepancies include failures in information submission, inconsistencies in system validations, delays in data updates, and, in some cases, underreporting (5, 6).

Data reliability and accurate analysis of notifications allow for the identification of epidemiological trends, assessment of intervention impacts, and more efficient resource allocation (7, 8). Without adequate and up-to-date data, public policies may be planned based on incomplete information, resulting in inefficient resource allocation, gaps in service coverage, and even failures in emergency response (9, 10). In this regard, territorial coverage and inequality in access to healthcare increase the complexity of data collection and transfer (2). Municipalities located in regions with poor infrastructure often face difficulties in implementing computerized registration systems (11). Therefore, the discrepancies between the values locally recorded in Sinan and those consolidated by DataSUS may highlight a structural issue that compromises the integrity of the health surveillance system.

Gaps in the database have direct implications for the accuracy of incidence, prevalence, and geographic distribution analyses of notified health conditions (10). For example, infectious disease outbreaks may be underestimated, hindering prompt interventions. Similarly, underreporting cases of violence or intoxications makes the implementation of integrated policies more difficult. From this perspective, this study evaluated whether there are quantitative differences between the health conditions recorded in Sinan and those consolidated in DataSUS, identifying potential database gaps. We hypothesized that there are differences in the number of notifications between the local system (Sinan) and DataSUS, based on the assumption that there is not an adequate flow of records collected by municipalities to the national system. This study reinforces the need to strengthen health information systems in Brazil, and in other countries, ensuring the proper consolidation of epidemiological data that support the development and direction of effective public policies.

## 2 Methods

In this ecological study conducted in Brazil, we used the municipality of Bragança, located in the Brazilian state of Pará, on the Amazon coast, as the study base. Brazil is divided into health regions, which are territorial divisions created to organize and decentralize the services of the Unified Health System (SUS) (12). These regions group together neighboring municipalities with similar characteristics and demands, allowing for the integration of healthcare networks. The state of Pará is divided into 13 health regions, with Bragança located in the Rio Caetés region. Bragança has approximately 131,000 inhabitants and a robust health network, with full coverage by the primary healthcare system. Among the most prevalent health conditions are transmissible infections such as syphilis, HIV, tuberculosis, leprosy, and viral hepatitis. In addition, waterborne diseases and arboviruses such as dengue require continuous surveillance and integrated actions within primary care. Bragança is one of the municipalities with the highest number of annual notifications, playing a strategic role in the epidemiological surveillance of this health region in the state of Pará.

### 2.1 Data collection

We obtained the Sinan dataset from the Bragança Municipal Health Department. Data were organized by health condition and by year of notification (2019–2023). We quantified only confirmed cases for each condition to allow comparison with DataSUS data. Notifications were filtered in the database according to the municipality of residence, excluding allochthonous cases that were sporadically notified in the municipality. Subsequently, we collected from DataSUS (https://datasus.saude.gov.br/informacoes-de-saude-tabnet/) the same health conditions notified in Sinan. This public repository from the Brazilian Ministry of Health consolidates information on the confirmed health conditions notified by Municipal Health Departments. The number of notifications in the database were filtered according to the municipality of residence (Bragança) by health condition and by year of notification (2019–2023). We did not include the year 2024 in the analysis because some conditions notified in the local system did not yet have corresponding information in DataSUS. The list of health conditions and the number of occurrences were requested from the Health Department of the municipality of Bragança. Furthermore, DataSUS data are anonymized and does not require ethical approval.

### 2.2 Data analysis

Data processing and analysis were conducted using GNU-R version 4.4.1 (13). Initially, we merged the Sinan and DataSUS databases and quantified the absolute loss in the number of notifications for each health condition and year. Due to the low number of observations per sample for the between-year and health condition (*n* = 5), we tested the assumptions of normality (Shapiro–Wilk test) and homogeneity (Levene test). If the data did not meet these assumptions, an appropriate non-parametric test would be applied. We formally tested the absolute loss in the number of notifications across years using a one-sample *t*-test (5% significance and 95% confidence) to verify whether the mean difference was statistically different from zero. The absolute loss for each condition was also tested using a one-sample *t*-test (5% significance and 95% confidence) to verify whether the mean difference was statistically different from zero. Subsequently, to visually assess the health conditions that showed differences in the number of notifications, we filtered the data by excluding conditions that were similar between Sinan and DataSUS over the years.

## 3 Results

A total of 19 health conditions and 3,461 notifications were notified in Sinan between 2019 and 2023 and were used to test our hypothesis (Tables 1, 2). These conditions were consistently reported throughout the period analyzed. In the same period, a total of 2,580 notifications were recorded in DataSUS for the same health conditions (Table 1). The assumptions of normality (Sinan: W = 0.87, *p* = 0.30; DataSUS: W = 0.91, *p* = 0.46) and homogeneity (*F* = 2.38, *p* = 0.42) were met, demonstrating the statistical power of the tests. Initially, when we analyzed the data by year (Table 1), we observed a significant absolute loss in the number of notifications between the two systems (*t* = 3.34; *p* = 0.01; mean [confidence interval] = 176 [118–233]). We observed that this loss was consistent from 2019 to 2023, with a reduction ranging from 37% to 42% (Table 1).

**Table 1:** Total number of notifications in Sinan and consolidated in DataSUS, and absolute and percentage (%) loss between the number of notifications by year.

**Year**	**Sinan**	**DataSUS**	**Absolute loss**	**% loss**
2019	765	517	248	42.5
2020	605	428	177	37.5
2021	639	514	125	40.5
2022	667	518	149	37.1
2023	785	603	182	42.1
Total	3,461	2,580	881	

**Table 2:** Total number of notifications in Sinan and consolidated in DataSUS, absolute loss in the number of notifications and mean losses with confidence intervals (CI) between the Sinan and the DataSUS, grouped by health conditions over the years (2019–2023); *t*-test values and *p*-values assessing differences in absolute loss for each condition across the years.

**Notified health conditions**	**Sinan**	**DataSUS**	**Absolute loss**	***t*-test; *p*-value**
AIDS	387	33	354	**8.4;** ** < 0.01**
Syphilis in pregnant women	251	109	142	**9.2;** ** < 0.001**
Dengue	200	71	129	**3.2; 0.03**
Meningitis	77	22	55	**4.6;** ** < 0.01**
Exanthematous diseases	105	55	50	1.3; 0.24
Viral hepatitis	58	26	32	**6.9;** ** < 0.01**
Tuberculosis	518	496	22	**3.42; 0.02**
Acquired syphilis	389	370	19	**4.7;** ** < 0.01**
Chagas disease	23	5	18	**3.1; 0.03**
Interpersonal/self-inflicted violence	389	371	18	1.0; 0.37
Leptospirosis	33	20	13	2.2; 0.09
Accidents involving venomous animals	235	223	12	**9.8;** ** < 0.001**
Chikungunya	178	167	11	1.3; 0.27
American cutaneous leishmaniasis	31	28	3	2.5; 0.07
Congenital syphilis	128	125	3	1.5; 0.20
Severe occupational accident	208	208	0	
Occupational accidents with biological material	102	102	0	
Leprosy	107	107	0	
Exogenous intoxication	41	41	0	

Bold values indicate statistically significant losses in the number of notifications.

**Figure 1:**
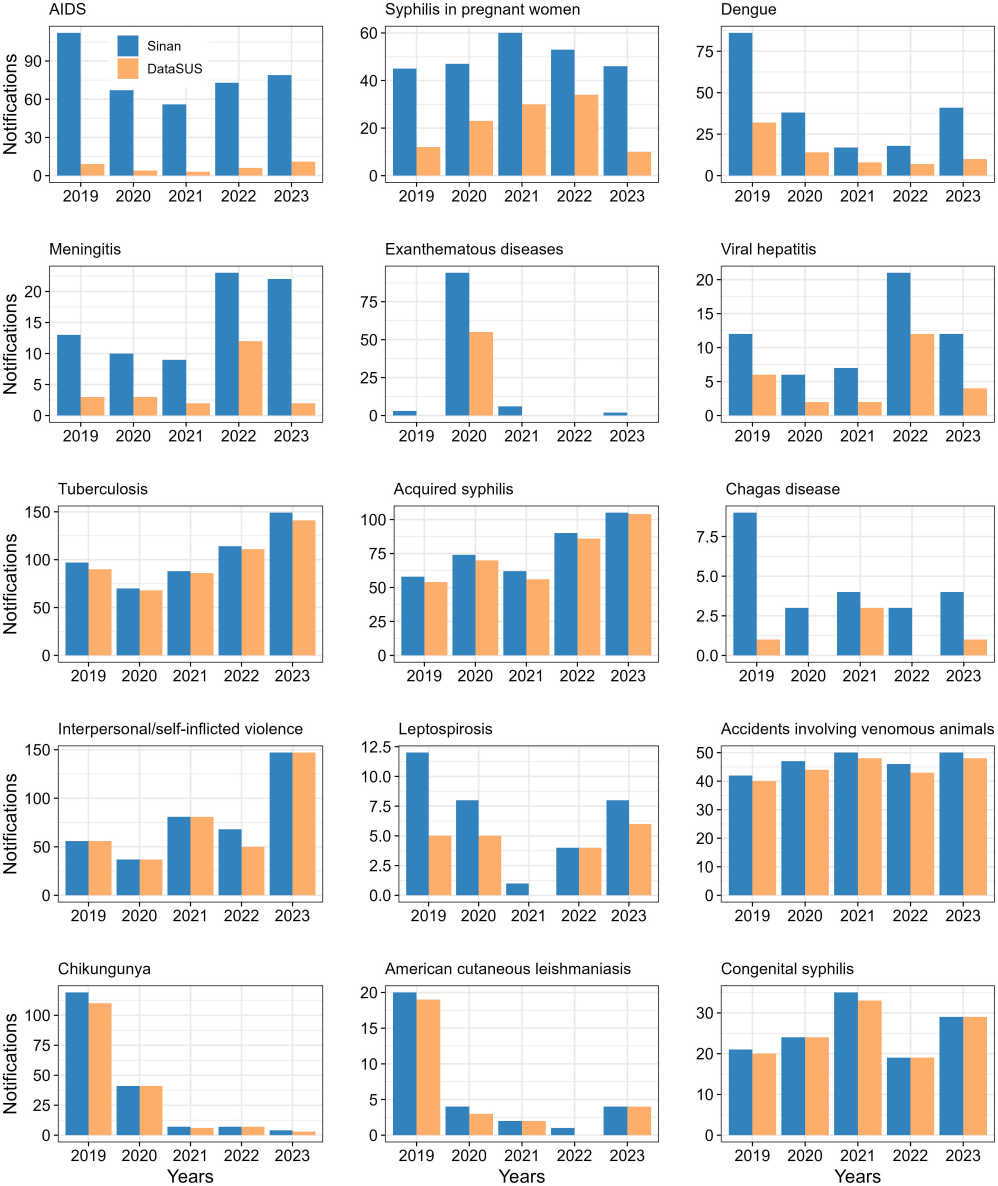
Total number of notifications in Sinan and consolidated in DataSUS for the notified health conditions with a loss in the number of cases in at least 1 year.

Fifteen conditions showed a loss in the number of notifications between Sinan and DataSUS in at least 1 year, and four were similar between the two systems over the 5-year period (Table 2). We observed a significant loss in the number of notifications between the two systems for nine conditions (see bold *p*-values in Table 2). The greatest losses were observed for notifications of AIDS, syphilis in pregnant women, and dengue (Figure 1). On the other hand, six conditions showed a loss in the number of notifications between the two systems, but these were not statistically significant (Table 2). Despite the large loss observed for exanthematous diseases, the greatest discrepancy occurred in 2020 (Figure 1). However, these notifications were recorded as an outbreak in Sinan and were not significant across the other years. The other conditions showed losses ranging from 3 to 55 notifications (Figure 1; Table 2).

## 4 Discussion

Our results revealed significant differences in the number of notifications between the Sinan and the DataSUS systems, supporting our hypothesis that there are differences between the two databases. Similar differences may also occur in other Brazilian municipalities, especially smaller ones (< 150,000 inhabitants), since all municipalities follow the same Sinan-DataSUS reporting protocol. However, this suggestion cannot be verified due to a lack of studies quantitatively analyzing information flows between Sinan and DataSUS databases. To our knowledge, this study is the first in Brazil to demonstrate these differences, providing an important baseline for revising health system data transmission protocols.

Other authors (1, 4, 5) suggest that the transition from paper-based epidemiological data to digital records must occur urgently and rapidly. This process should be associated with a standardized system and proper training of those responsible for local systems to avoid data loss (14). In other countries such as Honduras, Trinidad and Tobago, and Iraq, this transition is a recent process (7, 8, 11). Brazil has used computerized systems for approximately 40 years, which facilitates epidemiological analysis by the Ministry of Health. However, our findings suggest the occurrence of a serious issue that may be systemic and potentially compromise the development and execution of public health policies. This inconsistency and lack of accuracy in records may be associated with the distortion of epidemiological scenarios, which in turn hinders decision-making in various dimensions, including control, treatment, prevention, and the proper allocation of human, logistical, and economic resources.

The discrepancies between the two systems may not be random but associated with operational factors (15, 16). These inconsistencies may be interpreted as indicative of structural challenges in the Brazilian health ETL (Extract, Transform, Load) pipeline, as the system may experience breakdowns at multiple stages. During extraction, Sinan's protocol for capturing provisional diagnoses appears to conflict with DataSUS requirement for confirmed cases. In the transformation phase, diagnostic codes are often not fully reconciled, leading to mismatches such as Sinan generic HIV code B20 not aligning with DataSUS more specific B20.9. In the loading phase, undocumented validation rules automatically reject otherwise valid medical records based on technicalities rather than clinical criteria. These examples suggest that data discrepancies may be better understood as stemming from structural and policy-level features of the national health information system. Thus, they are unlikely to result from local implementation problems or technical shortcomings that ETL specialists could resolve through standard data management practices.

Deficiencies in data collection may hinder integration between decision-making bodies (5). For example, the loss of outbreak notifications, as observed for exanthematous diseases in 2020, could have contributed to temporary data inconsistencies, potentially delaying vaccination blocking plans and the distribution of supplies. Similarly, gaps suggested by our results for AIDS or syphilis in pregnant women may lead to an underestimation of cases, potentially compromising resource allocation and the implementation of effective interventions (3). Moreover, such underreporting could result in delays in early diagnosis and treatment, increasing the risk of vertical transmission and neonatal complications. Gaps in notifications for conditions like dengue may impair early outbreak detection and could hinder the implementation of vector control measures in specific areas, such as a neighborhood or rural community.

To address these systemic challenges, it is important to adopt measures that include training health professionals, reviewing notification workflows (17), and investing in integrated technologies that facilitate more efficient integration of databases (18, 19). We propose implementing a data validation framework with three core components: real-time validation protocols using automated checks and cryptographic tracking during data entry; unified training programs aligned with Brazilian National Health Policy, incorporating mandatory certification and regular audits; and technical integration to enable seamless synchronization between systems. This framework builds on successful pilots in Brazil for canine visceral leishmaniasis (18) and maternal health (19), where similar measures reduced reporting discrepancies by 35–40% within 1 year. Integrated frameworks in other countries have also proven effective (17, 20). By shifting from reactive corrections to preventive quality control, the proposed approach addresses root causes of data inconsistency while remaining adaptable to other low-resource settings. Its implementation would not only improve the reliability of epidemiological data but also enhance the effectiveness of public health policies that depend on accurate surveillance information.

## 5 Conclusion

Our results highlight the need to improve the quality of epidemiological surveillance systems by reducing the gaps between local and national systems. The differences observed between the two databases may have direct implications for the development and successful execution of public health policies. Implementing strategies for health professional training, reviewing notification workflows, and investing in technologies that facilitate system integration are likely to be important measures for improving consistency and accuracy. In addition, our findings in Bragança may reflect systemic challenges affecting many Brazilian municipalities, as all follow the same standardized Sinan-DataSUS reporting protocol. While local infrastructure and population characteristics may vary, the fundamental data transmission workflow, from frontline health unit documentation to municipal compilation and eventual national consolidation, remains largely consistent across Brazilian unified health system. The discrepancy patterns we observed, particularly for conditions requiring complex diagnostics, may occur nationwide as they plausibly stem from inherent tensions in the system design: the need for rapid local reporting vs. rigorous national validation standards. The issues identified, including code mapping inconsistencies and validation timing gaps, may represent structural challenges in Brazilian health information architecture rather than unique local phenomena. Although this study did not directly assess the impact of data discrepancies between Sinan and DataSUS, such inconsistencies could have important implications for public health programming. Incomplete or inaccurate data might compromise disease surveillance, hinder timely decision-making, and affect the allocation of resources. The loss of information may reduce the effectiveness of health interventions and impair efforts to monitor and control communicable diseases at the population level. Future research should investigate these impacts in greater detail to guide strategies for improving data quality and integration within national health information systems.

## Data Availability

The raw data supporting the conclusions of this article will be made available by the authors, without undue reservation.
